# Paroxysmal nocturnal hemoglobinuria‐phenotype cells predict a good response to eltrombopag in patients with refractory aplastic anemia

**DOI:** 10.1002/jha2.51

**Published:** 2020-06-29

**Authors:** Ken Ishiyama, Keijiro Sato, Tatsuya Imi, Kohei Hosokawa, Yukio Kondo, Naomi Sugimori, Hirohito Yamazaki, Shinji Nakao

**Affiliations:** ^1^ Department of Hematology Kanazawa University Hospital Ishikawa Japan; ^2^ Department of Hematology Nagano Red Cross Hospital Nagano Japan

**Keywords:** aplastic anemia, PNH, thrombopoietin

## Abstract

To identify factors affecting responsiveness to eltrombopag (EPAG), we retrospectively analyzed 38 aplastic anemia patients treated with EPAG who were refractory (n = 29) or showed an inadequate response (n = 9) to conventional therapies. The efficacy was evaluated at 16 weeks after starting EPAG and at any given time when the best response was achieved. Hematologic responses were observed in 15 patients (39%) at week 16 and in 25 (66%) at any given time. Ten of 19 (53%) achieved transfusion independence. A univariate analysis revealed the presence of PNH‐phenotype cells and the relatively higher platelet counts as associated with a good response to EPAG.

## 1 INTRODUCTION

Although the first‐line treatment for patients with acquired aplastic anemia (AA) is immunosuppressive therapy (IST), approximately one‐third of patients do not respond to IST. Eltrombopag (EPAG) has been shown to be effective in more than 40% of patients with AA refractory to conventional therapies [1]. However, which patients benefit from EPAG remains unclear.

To characterize AA patients who are likely to respond to EPAG, we studied 38 patients with refractory AA treated with EPAG.

## 2 PATIENTS AND METHODS

Of the 38 patients (severe 17, non‐severe 21; 21 to 86 years old, median 52 years old; 19 transfusion‐dependent), 29 had not responded to various therapies and had remained pancytopenic for more than 1 year (RAA), and nine had only shown an inadequate response to IST (IrAA), which was defined as a reduction for red blood cell transfusions, improvement of blood counts, and not meeting the response criteria according to the previous publication by Gluckman et al [2] (Table 1a).

**TABLE 1:** **(a)** Patient characteristics. **(b)** Response to EPAG. **(c)** Comparison between the responders and non‐responders to EPAG

(a)		All patients (n = 38)	RAA (n = 29)	IrAA (n = 9)
Age, median (range)		52 (21‐86)	50 (21‐86)	53 (31‐64)
Gender	Female/male	21/17	14/15	7/2
Median months from diagnosis to EPAG		130 (10‐470)	157 (10‐470)	111 (14‐274)
Stage at starting EPAG	Non‐severe/severe	28/10	20/9	8/1
History of IST	Yes/no	31/7	23/6	8/1
PNH‐phenotype cells	Positive/negative	8/29	5/23	3/6
Laboratory data at starting EPAG	WBC (10^9^/L)	2400 (1030‐5000)	2400 (1030‐5000)	2350 (1080‐4140)
	neu (10^9^/L)	1010 (160‐4050)	945 (160‐4050)	1195 (470‐2580)
	Hb (g/L)	7.7 (4.1‐14.6)	7.0 (4.1‐12.3)	10.4 (5.3‐14.6)
	plt (10^9^/L)	1.8 (0.6‐7.3)	1.8 (0.6‐5.0)	3.3 (1.0‐7.3)
	#ret (10^9^/L)	4.3 (0.4‐13.0)	4.3 (0.4‐9.7)	4.2 (0.7‐13.0)
	WT1 mRNA (copies/μgRNA)	88 (< 50‐350)	88 (< 50‐350)	93 (< 50‐210)
Karyotype at starting EPAG	Trisomy 8	4	4	0
	t(6;12)(q23;p13)	1	0	1
	del(13)(q12q14)	1	0	1
	Normal	27^*^	21^*^	6

(b)	At 16 weeks			Best overall response		
	All patients (n = 38)	RAA (n = 29)	IrAA (n = 9)	All patients (n = 38)	RAA (n = 29)	IrAA (n = 9)
Responding ≧ 1 lineage	39% (15/38)	34% (10/29)	56% (5/9)	66% (25/38)	66% (19/29)	67% (6/9)
Erythrocyte	30% (8/27)	33% (8^*^/24)	0% (0/3)	70% (19/27)	71% (17^†^/24)	67% (2/3)
Platelet	21% (8/38)	10% (3/29)	56% (5/9)	42% (16/38)	38% (11/29)	56% (5/9)
Neutrophil	5% (1/19)	0% (0/16)	33% (1/3)	32% (6/19)	31% (5/16)	33% (1/3)

(c)		At 16 weeks			Best overall response		
		Responder (n = 15)	Non‐responder (n = 23)	*P*‐value	Responder (n = 25)	Non‐responder (n = 13)	*P‐*value
Age, median (range)		50 (21‐86)	57 (21‐72)	.39	55 (21‐86)	49 (21‐72)	.79
Gender	Female/male	8/7	13/10	1	11/14	10/3	.086
Median months from diagnosis to EPAG		119 (10‐470)	143 (10‐459)	.26	119 (10‐470)	143 (24‐445)	.26
Stage at starting EPAG	Non‐severe/severe	9/6	12/11	.74	15/10	6/7	.5
History of IST	Yes/no	12/3	19/4	1	21/4	10/3	.67
PNH‐phenotype cells	positive/negative	5/10	3/19	.23	8/16	0/13	**.032**
Laboratory data at starting EPAG	WBC (10^9^/L)	2300 (1270‐4160)	2700 (1030‐5000)	.41	2400 (1270‐4160)	2400 (1030‐5000)	.87
	neu (10^9^/L)	810 (250‐2580)	1124 (160‐4050)	.22	945 (250‐2580)	1128 (160‐4050)	.7
	Hb (g/L)	8.2 (5.3‐14.6)	7.6 (4.1‐13.9)	.33	7.2 (5.3‐14.6)	8.0 (4.1‐13.9)	.82
	plt (10^9^/L)	3.2 (0.9‐7.3)	1.8 (0.6‐6.5)	**.013**	2.0 (0.6‐7.3)	1.8 (0.6‐6.5)	.24
	#ret (10^9^/L)	4.3 (0.7‐13.0)	4.4 (0.4‐9.7)	.92	4.3 (0.7‐13.0)	3.9 (0.4‐9.7)	.26
	WT1 mRNA (copies/µgRNA)	87 (<50‐230)	99 (<50‐350)	.52	69 (<50‐350)	99 (<50‐250)	.21

* The following abnormalities were observed in 1 of 20 cells; 1 patient with trisomy 8 and 1 patient with monosomy 7.

Secondary refractory patients were included as follows; ^*^1 patient, ^†^2 patients.

^a^Efficacy evaluated at 16 weeks.

^b^Efficacy evaluated as best overall response.

Abbreviations: EPAG, eltrombopag; Hb, hemoglobin; IrAA, aplastic anemia inadequate response to immunosuppressive therapy; IST, immunosuppressive therapy; neu, neutrophil; plt, platelets; PNH, paroxysmal nocturnal hemoglobinuria; RAA, aplastic anemia refractory to conventional therapies; ret, reticulocyte; UPN, unique patient number.

The efficacy was evaluated at 16 weeks after starting EPAG and at any given time when patients achieved response (best overall response). The response was defined according to the criteria by Desmond et al.[3] EPAG was continued in non‐responders at 3 months to anticipate further improvement when the patients showed some signs of improvement that did not meet the response criteria.

The clinical characteristics of the patients who responded and those who failed to respond to EPAG were compared using Fisher's exact test for categorical variables and the *t*‐test for continuous variables. *P*‐values were two‐sided, and outcomes were considered significant when *P*‐values were <.05. All statistical analyses were performed using EZR (Saitama Medical Center, Jichi Medical University), which is a graphical user interface for the R software program (The R Foundation for Statistical Computing; http://www.r-project.org, version 3.3.2).[4] Adverse events developing during EPAG therapy were evaluated according to the Common Terminology Criteria for Adverse Events (CTCAE) of the National Cancer Institute, USA, ver.4.0. This study was approved by the ethics committee of Kanazawa University (Study #2779).

## 3 RESULTS

### 3.1 Patients’ characteristics

The median duration from the AA diagnosis to the start of EPAG treatment was 130 (range: 10‐470) months. The maximal doses of EPAG were 100 mg/day in 30 patients and 50‐75 mg/day in eight patients. Thirty‐one patients had received IST prior to EPAG. The median reticulocyte count was 4.3 × 10^9^/L (range: 0.4‐13.0 × 10^9^/L). The numbers of patients who required regular blood transfusions were 14 for red blood cells, 4 for both red blood cells and platelets, and 1 for platelets. Eight (5 with RAA and 3 with IrAA) patients possessed 0.056‐97.879% (median: 0.994%) glycosylphosphatidylinositol‐anchored protein‐deficient (paroxysmal nocturnal hemoglobinuria [PNH]‐type) cells, which are known to be associated with a good response to IST. Chromosomal abnormalities were detected in four (trisomy 8) of the 25 RAA patients and two (t(6;12)(q23;p13) and del(13)(q12q14)) of the eight IrAA patients.

### 3.2 Response to EPAG

Fifteen of 38 (39%) patients (34% of RAA patients and 56% of IrAA patients) showed a hematologic response in at least one lineage at 16 weeks (Table 1b). The best overall response rate was 66% (66% [19/29] in RAA and 67% [6/9] in IrAA patients, and 75% [21/28] in NSAA and 40% [4/10] in SAA patients, respectively). Eight of 16 (50%) RAA patients and two‐third (67%) IrAA patients achieved transfusion independence.

**FIGURE 1:**
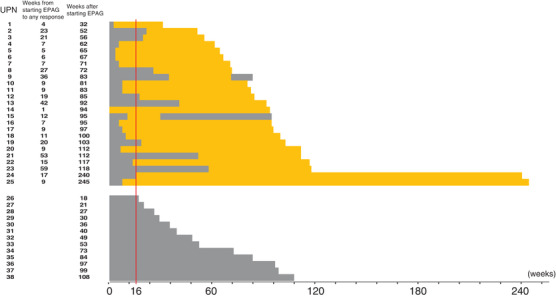
Treatment duration and response to EPAG. Solid‐orange bars and solid‐gray bars indicate responders and non‐responders to EPAG. Secondary refractoriness to EPAG was observed in two patients; the time to the response and the duration of the response in these two cases were 36 weeks and 36 weeks in UPN 9, and 12 weeks and 19 weeks in UPN 15, respectively

The median time from starting EPAG to a response in at least one lineage was 9 weeks (range: 1‐80 weeks, Figure 1). The median duration of EPAG administration was 92 weeks (range: 32‐245) in responders and 49 weeks (range: 18‐108) in non‐responders. Five of the six patients with chromosomal abnormalities responded without an increase in the percentage of abnormal clones. Secondary refractoriness to EPAG was observed in two patients; the time to the response from the start of EPAG and the duration of the response in these two cases were 36 weeks and 36 weeks in UPN 9, and 12 weeks and 19 weeks in UPN 15, respectively.

Fourteen adverse events were observed in 12 patients during EPAG treatment (Table S1); all events were grade 1 and were improved by reducing the dose of EPAG. In one non‐responder to EPAG (UPN 33), the percentage of trisomy 8‐positive granulocytes as determined by a fluorescence in situ hybridization analysis increased from 5% to 20.4% after 25 months of EPAG. No patients showed any new chromosomal abnormalities or an increase in the WT1 mRNA copy number of peripheral blood or progression to clonal disorders, including myelodysplastic syndromes and PNH.

### 3.3 A comparison between responders and non‐responders to EPAG

In a univariate analysis, the platelet count (0.9‐7.3 × 10^9^/L, median 3.2 × 10^9^/L) at the start of EPAG in 15 responders at 16 weeks was significantly higher than that (0.6‐6.5 × 10^9^/L, median 1.8 × 10^9^/L) in 23 non‐responders (*P* = .013, Table 1c). When the response was evaluated according to the best overall response, the response rate in eight patients positive for PNH‐phenotype cells (PNH[+], 100%) was significantly higher than that (55%) of the patients (16/29) negative for PNH‐phenotype (*P* = .032). In addition, male patients tended to respond to EPAG better than female patients (*P* = .086). There were no marked differences in the age, time from the diagnosis to the start of EPAG, number of treatment regimens prior to EPAG, neutrophils/Hb/reticulocytes or WT1 mRNA copy number at the start of treatment between the responders and non‐responders.

## 4 DISCUSSION

There are only a few reports on the efficacy of EPAG in patients with RAA and IrAA based on large cohorts. Olnes et al.(n = 25) and Desmond et al (n = 43) reported that higher reticulocyte counts at the start of EPAG were a predictor of the EPAG response.[5],[3] Although no such tendency was observed in our study, higher platelet counts and PNH‐phenotype cells at the start of EPAG were factors for predicting the EPAG response. Small populations of PNH‐phenotype cells are detected in approximately 50% of patients with newly diagnosed AA,[6] and are thought to represent benign bone marrow failure.[7] In this study focusing on refractory AA cases, such PNH(+) patients accounted for only 22% of our population. The favorable response to EPAG in PNH(+) patients suggests that even though they failed to respond to IST, hematopoietic stem/progenitor cells of PNH(+) patients may be healthier than those of patients without increased PNH‐phenotype cells. Fattizzo et al. reported a higher response rate to EPAG in 32 PNH(+) cases than in 17 PNH(‐) cases. However, the study included seven treatment‐naive patients, and the response rate to EPAG in their 42 relapse/refractory patients was 15%.[8] It is unclear how many responders in the relapse/refractory cases had PNH clones. Our study is therefore first to demonstrate a predictive value of PNH clones for a good response to EPAG in RAA or IrAA patients.

In this study, the maximum dose of EPAG was 100 mg, which is equivalent to 200 mg for non‐East Asian subjects [9]. The therapeutic effect of EPAG was evaluated at 2 points: 16 weeks and at any given time when patients achieved the best response. While the Japanese medical insurance system recommends that physicians decide whether or not to continue EPAG by week 16 after starting the treatment, in the present study, 40% of patients responded to EPAG at 17 weeks or later. When the laboratory data at the start of EPAG treatment were compared between 15 responders at 16 weeks and 10 responders at 17 weeks or later, the platelet counts in the former group was significantly higher than that in the latter (Table 1c.; 32.0 × 10^9^/L vs 18.0 × 10^9^/L, *P* = .013). These findings support the report from NIH that the prolonged administration of EPAG is needed to achieve response in a cohort of SAA patients.[10] Current study describes the ability to predict response to EPAG, based on higher platelet counts, which is also a reflection of severity of the disease, for instance, less severe and more chance to respond. Among the six patients whose platelet counts at the start of EPAG was <10.0 × 10^9^/L, three (50%) responded by 19 weeks, while none showed a response beyond 20 weeks from the start. Based on these findings, changing the treatment may need to be considered if patients with platelet counts <10.0 × 10^9^/L at the start of EPAG do not show signs of a response by 20 weeks.

## 5 CONCLUSIONS

Long‐term EPAG treatment was effective in approximately 66% of RAA patients and 67% of IrAA patients. In the 29 RAA patients, the best overall response rate was 75% in NSAA patients and 44% in SAA. The presence of PNH‐phenotype cells and relatively high platelet counts may represent the markers for good response to EPAG.

## AUTHOR CONTRIBUTIONS

KI, KS, and SN designed studies; TI, YK, NS, HY, and SN provided clinical care; TI and KH performed laboratory studies; KI and KS analyzed the data; KI, KS, and SN performed statistical analyses; KI and SN wrote and edited the manuscript.

## CONFLICT OF INTEREST

KI, HY, and SN received honoraria fees and lecture fees from Novartis Pharma K.K.

## Supporting information


**Supplemental Table 1**. Adverse events during EPAG treatment.
Abbreviations: CTCAE, common terminology criteria for adverse events; EPAG, eltrombopag.
