# Supplementary material for: Paroxysmal nocturnal hemoglobinuria‐phenotype cells predict a good response to eltrombopag in patients with refractory aplastic anemia
Source: EJHaem. 2020 Jun 29;1(1):243–8. doi: 10.1002/jha2.51 (PMC9175921; doi:10.1002/jha2.51)
Supplement: Supplementary file 1 — Supplemental Table 1. Adverse events during EPAG treatment. Abbreviations: CTCAE, common terminology criteria for adverse events; EPAG, eltrombopag. [file JHA2-1-243-s001.pdf]

Supplemental Table 1. Adverse events during the EPAG treatment.

CTCAE term	Frequency (#)
liver dysfunction	11% (4/38)
pigmentation	5% (2/38)
fatigue	5% (2/38)
renal dysfunction	5% (2/38)
fever	3% (1/38)
lumbago	3% (1/38)
nausea	3% (1/38)
tremor	3% (1/38)

All of the events above were grade 1.
